# Siwa Oasis groundwater quality: factors controlling spatial and temporal changes

**DOI:** 10.1007/s10661-022-10646-z

**Published:** 2022-11-03

**Authors:** Ahmed A. Elnazer, Salman A. Salman, Yasser M. A. Mohamed, Jason Stafford, Philip Davies, Hossam A. El Nazer

**Affiliations:** 1 Geological Sciences Department, National Research Centre, 33 El Bohouth St. (Former El-Tahrir St.), Dokki, Giza, Egypt; 2 Photochemistry Department, National Research Center, Dokki, Giza, 12622 Egypt; 3 School of Engineering, University of Birmingham, Edgbaston, Birmingham, B15 2TT UK

**Keywords:** Groundwater, Salinity, Temporal changes, Heavy metals, Siwa

## Abstract

Siwa Oasis is of great historical, environmental, and scientific importance, as it contains unique archeological and geological features. Groundwater is the main source of freshwater in that oasis. The carbonate aquifer groundwater, used for irrigation, was sampled to evaluate factors controlling quality changes spatially and temporally by applying hydrochemical and statistical analyses. The salinity of the aquifer varied spatially from 1367 to 8645 mg/l based on one hydrogeological condition, with the highest TDS (> 5432.5 mg/l, 25% of samples) at the central part of the study area. Temporally, the salinity changed slightly from 3754.3 mg/l (in 2014) to 4222.4 mg/l (in 2020). The cession of illegal wells, pumping control, and excavation of formed salts have a noticeable impact on salinity (mediate the increase in salinity) and ions. However, about 61% of the studied samples can be considered unsuitable for irrigation owing to salinity and can harm plant yield. The heavy metals studied (Fe, Mn, Cu, Pb), except Cd, were within the permissible limit for irrigation water. Finally, it is proposed to construct desalination stations to enhance water quality for irrigation in the study area and set up many companies for salt extraction.

## Introduction

Siwa Oasis is the oldest oasis in Egypt, represents the last virgin oasis in the western desert of Egypt, and is a depression located 1–20 m below sea level. Siwa Oasis is surrounded by rocky hills from the north, the Great Sand Sea in the south, and four salty lakes in the middle of the depression. The Siwa oasis is one of the important agro-ecosystems in the Western Desert that mainly depends on groundwater for irrigation and drinking purposes (Aly & Benaabidate, 2010).

Groundwater in the Arab Republic of Egypt is distributed among several renewable and non-renewable groundwater basins, some of which have been drained by unbalanced withdrawals, which led to changes in the quantity and quality of water, high salinity rates, in addition to the high cost of withdrawal. As a result, there has been a great decline in groundwater levels. Siwa Oasis has many natural springs and shallow and deep wells. The number of springs scattered in the oasis now reaches about 200, of which only about 80 are used for irrigation or drinking; they are called “Roman eyes.” Some springs are used in medical treatment (sulfur springs), the most famous of which is Ain Cleopatra (Al-Damiri, 2005). More than 1200 wells extract water from the shallow (with depth 10–200 m) limestone aquifer, characterized by its high saline (TDS up to 8000 mg/l) water. In contrast, the deep Nubian sandstone aquifer contains freshwater with TDS < 256 mg/l (Aly, 2015). The average daily discharge of wells from these aquifers is 900 × 10^3^ m^3^/day (Abdulaziz & Faid, 2015; FAO, 2016). The use of saltwater for irrigation will affect the ability of plants to absorb water and nutrients owing to the increase of osmosis pressure around plant roots (Elhindi et al., 2020). The uncontrolled withdraw of groundwater and agricultural expansion in the last decades has led to the decline of the piezometric head levels and the deterioration of groundwater quality (Salman et al., 2018).

Groundwater has a special interest in arid regions owing to its importance as a unique source of freshwater for different purposes. In such regions, groundwater has the main impact on social, agricultural, and industrial sustainable development projects. Groundwater quality has a significant impact on ecologic constituents, soil quality and plant growth and yield, and consequentially human health (Gupta, 2012; Ramos-Leal et al., 2016). Mostly, the previous studies dealt with the hydrochemical evaluation of the groundwater resources in the Oasis (e.g., Hassan & Ismail, 2018; Moghazy & Kaluarachchi, 2020; Salman et al., 2018) and point out the salinization process of the limestone aquifer. This study aimed to investigate the carbonate aquifer hydrochemistry (spatial and temporal changes), factors controlling groundwater geochemistry, suitability for irrigation, and the impact of human activities such as environmental and hydrological precaution affect groundwater quality.

## Materials and methods

### Hydrogeological settings

**Fig. 1:**
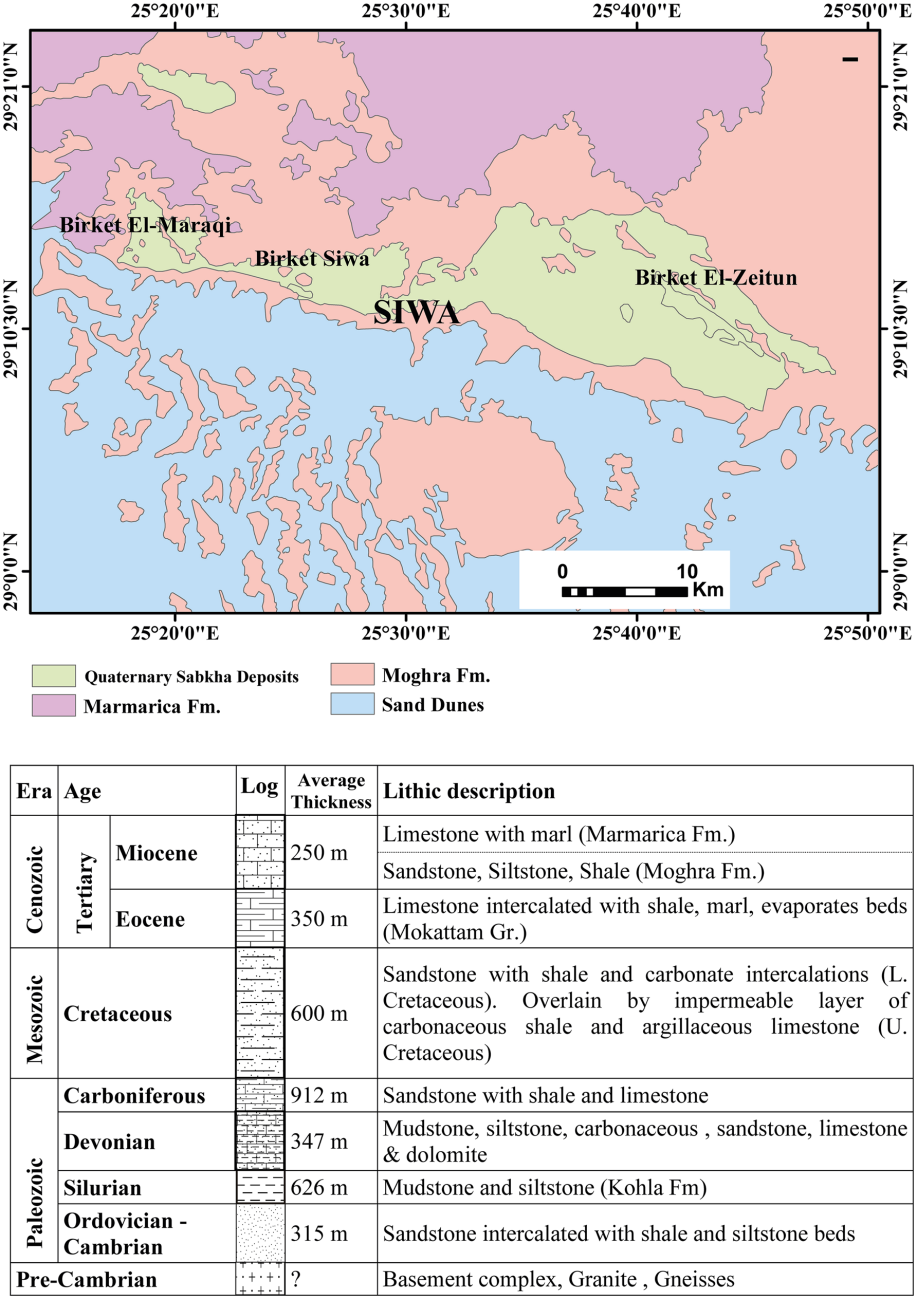
Geologic map of Siwa oasis (modified CONOCO, 1986) and geologic cross-section (after Abdel-Gawad et al., 2020; El-Sayed et al., 2017)

Siwa Oasis is considered the virgin and smallest oasis of the seven major important natural depressions located in the Western Desert of Egypt, where it sits in a closed structural eroded deep depression that reaches below sea level and varies in altitude between ~ 1 and − 18 m. It lies between latitudes 29°05′00″ N and 29°19′00″ N and longitudes 25°12′00″ E and 25°55′00″ E (Fig. 1). A desert climate (arid to semi-arid conditions) characterized the Siwa Oasis, where it displays scarce rainfall, a short and mild winter season, and a long hot and dry summer (Abdel-Gawad et al., 2020). Geologically, Siwa Oasis contains a thick (3400 m) sedimentary sequence ranging from the Paleozoic to the Recent affected by many normal faults (N-S, E-W, NE-SW, and NW–SE). The surface of the oasis is covered mainly by the sabkhas (silt, clay, and evaporates deposits) and sand dunes of the Quaternary age (Fig. 1). Also, the Middle Eocene–Mokattam Group is exposed in the SE part of the oasis and composed of white chalky limestone, gray shale, and neritic limestone beds (Afifi, 2005; Said, 1962). The structure lines control lithofacies variation, formations thicknesses, and the appearance of natural springs (Shata, 1982).

Hydrologically, Siwa contains two main aquifers: the Nubian sandstone aquifer (NSA) and the Tertiary carbonate confined aquifer (TCCA) (Dahab, 2004; El Hossary, 2013). The Eocene–Miocene formations is composed of limestone and dolomite intercalated with shale, siltstone, sandstone, and evaporate deposits (Fig. 1), with an average thickness of 550 m (El Hossary, 2013). The upward seepage of water from the NSA is the main source of TCCA recharge (Dahab, 2004). The discharge of the aquifer is through the wells and natural springs with a rate that reaches 442000 m^3^/day and with a great spatial variation in water quality based on the variation in the penetrated lithofacies (Abdel-Mogheeth, 1996).

### Sampling and analyses

**Fig. 2:**
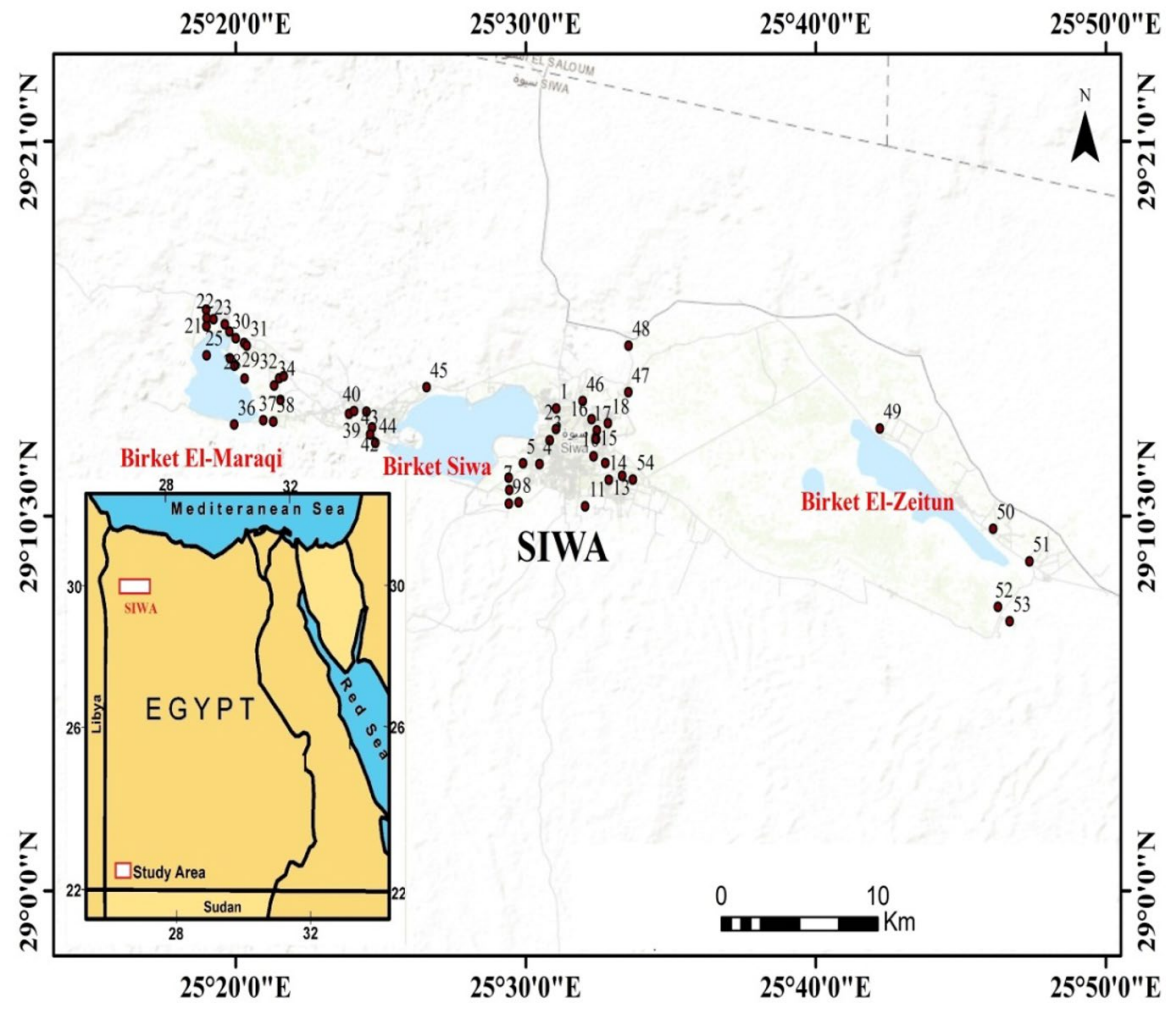
Map showing the study area and sampling wells

Fifty-four samples of groundwater were collected randomly from artesian wells in Siwa Oasis, Egypt (Fig. 2), based on literature survey, accessibility, and safety. The samples were selected to cover the studied aquifer and surround the lakes and from different farms as accessible. The samples were placed in pre-washed polypropylene bottles and closed tightly. The parameters of pH, TDS, temperature, and electrical conductivity (EC) were determined in situ using a digitally combined electrode (HANNA HI 991300). Redox potentiality (Eh) was measured in situ using a portable electrode (Hanna HI 98120). Samples were filtered in the laboratory using a 45-µm filter and were analyzed for chemical constituents according to APHA standard procedures (1995). Whole water analysis was performed 1 day after sampling and storage at 4 °C. A flame photometer was used to determine both Na and K. Volumetric methods were used to measure Ca, Mg, CO_3_, HCO_3_, and Cl. A spectrophotometer (HANNA HI 83215) was used to measure the SO_4_ and NO_3_.

#### Sodium absorption ratio (SAR)

Sodium absorption ratio was calculated to determine the suitability of water for irrigation according to Richards (1954) equation (all values in meq L^−1^):

$$SAR={Na}^{+}/{[({Ca}^{2+}+{Mg}^{2+})/2]}^{1/2}$$

ArcGIS 10.4.1 was used for the resulting distribution maps. To create spatial distribution maps of elements within the study area, the inverse distance weighting (IDW) was used. This technique is useful for understanding and predicting pollutant spread and for conducting environmental monitoring.

The salinization process was evaluated by Revelle (1941) index (RI) (Eq. 1). Groundwater with RI values > 0.5 is considered to be affected by salinization (Karunanidhi et al., 2020). Chloro-alkaline indices CAI-1 (Eq. 2) and CAI-2 (Eq. 3) (Schoeller, 1965) were applied to deduce ion exchange activity in groundwater. Positive CAI-1 and CAI-2 are indicative of reverse ion exchange reaction where Na and K replace Ca and Mg ions, leading to an increase of Ca and Mg concentration in groundwater. In contrast, this becomes a forward ion exchange reaction when CAI-1 and CAI-2 values are negative with Ca and Mg replacing Na and K leading to the increase of Na and K concentration in groundwater. Moreover, if CAI-1 and/or CAI-2 are zero, it indicates that the ion exchange does not occur during the formation of the groundwater. Kaur et al. (2019) mentioned that the larger the absolute values of (CAI-1 and CAI-2) are, the stronger the ion exchange interaction is:

1 $$RI=C1/({HC0}_{3}+{CO}_{3})$$

2 $$CAI-1=(C1-(Na+K))/C1$$

3 $$CAI-2=(C1-(Na+K))/({SO}_{4}+{HCO}_{3}+{CO}_{3}+{NO}_{3})$$

### Statistical analysis

All statistical analyses were performed using SPSS 16.0 software. Descriptive statistics were conducted to understand the distribution of groundwater parameters. To highlight the main process controlling water composition, PCA was performed using Varimax rotation. Kaiser–Meyer–Olkin measure of sampling adequacy (KMO), with Bartlett’s test for sphericity, was applied.

## Results and discussion

### Hydrochemical characteristics

**Fig. 3:**
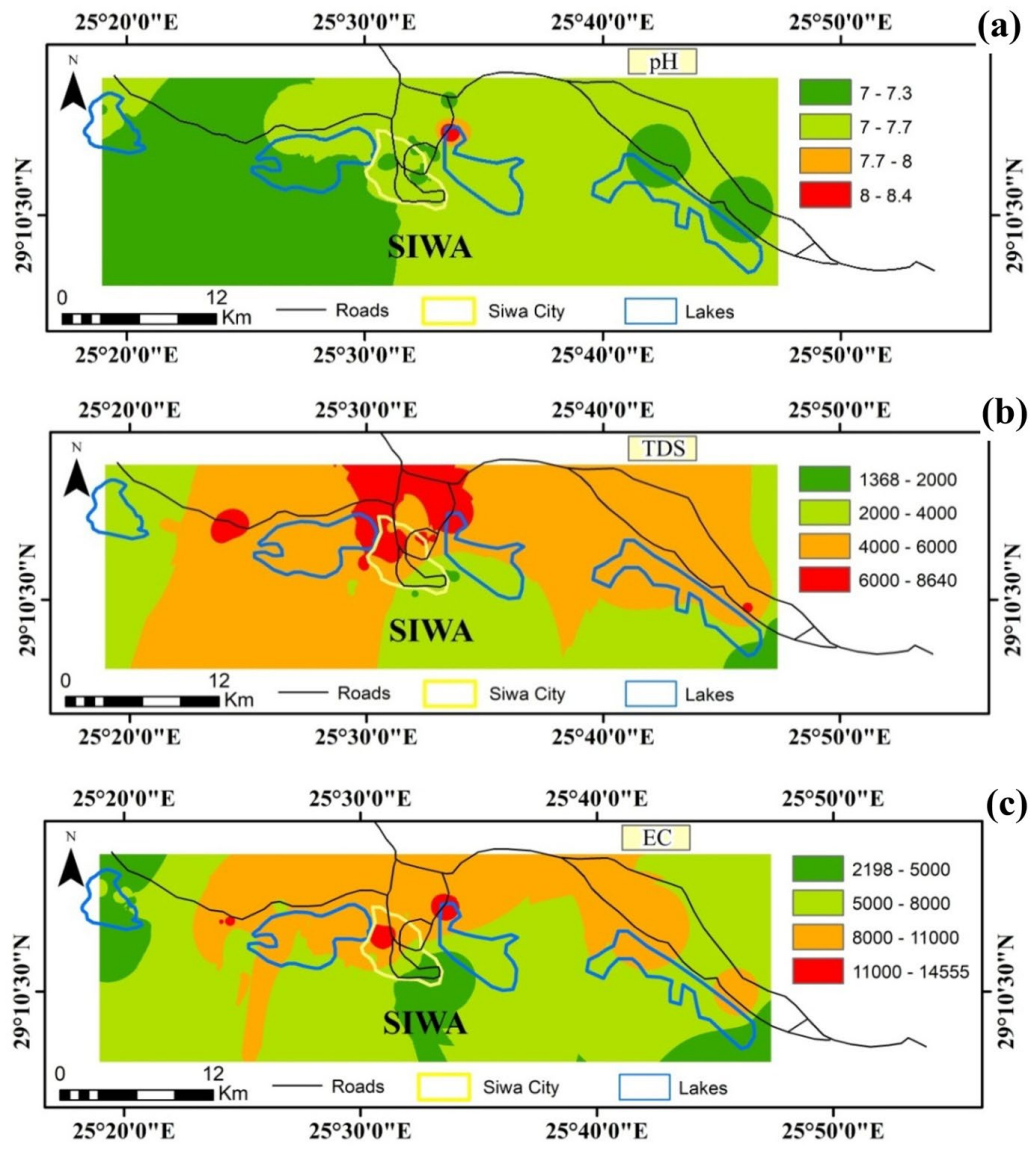
Spatial distribution of **a** pH, **b** TDS (mg/l), and **c** EC (µS/cm) in the study area

The descriptive statistics of the studied hydrochemical parameters were illustrated in Table 1. The studied groundwater is slightly alkaline with a pH value ranging from 7.01 to 8.44. According to the USDA (2011), all the water samples are within the preferred limit, 6.5–8.5 for irrigation. A very high distribution of pH (high alkalinity) is near the central salt lake of the study area due to high activities and salt fabrication (Fig. 3a).

**Table 1:** Descriptive statistics of the studied hydrochemical parameters

	**pH**	**T**	**Eh**	**TDS**	**EC**	**Ca**	**Mg**	**Na**	**K**	**HCO**_**3**_	**Cl**	**SO**_**4**_	**NH**_**4**_	**NO**_**3**_	**Fe**	**Mn**	**Cu**	**Pb**	**Cd**	**RI**	**CAI-1**	**CAI-2**
Unit	-	°C	mV	mg/l	µS/cm	mg/l									µg/l					-	-	-
Mean	7.32	29.8	−29.1	4222	6672	201.2	148.6	1141.8	34.0	141.5	1917.5	761.1	0.46	1.38	2359.4	898.8	448.9	865.8	52.8	24.9	0.1	0.3
Median	7.28	29.8	−38.0	3802	5561	196.0	129.6	955.0	30.9	141.5	1722.0	601.9	0.50	1.34	2281.5	323.0	476.5	702.5	43.5	21.6	0.1	0.3
SD	0.21	2.0	90.0	1842	3141	72.9	64.7	660.4	13.2	30.1	961.3	482.8	0.14	0.56	1008.3	1074.2	237.7	795.2	38.2	15.1	0.1	0.3
Range	1.43	9.2	417.0	7277	12,363	248.0	240.0	2712.2	80.2	165.9	3833.2	2009.1	0.53	2.94	3889.0	3901.0	748.0	3080.0	245.0	80.9	0.5	1.6
**Min**	**7.01**	**25.0**	** −213.0**	**1367**	**2198**	**72.0**	**33.6**	**292.6**	**9.4**	**68.3**	**462.0**	**114.8**	**0.17**	**0.42**	**755.0**	**0.0**	**80.0**	0.0	15.0	5.1	−0.2	−0.4
**Max**	**8.44**	**34.2**	**204.0**	**8645**	**14,561**	**320.0**	**273.6**	**3004.7**	**89.5**	**234.2**	**4295.2**	**2123.9**	**0.70**	**3.36**	**4644.0**	**3901.0**	**828.0**	3080.0	260.0	86.0	0.3	1.3
Q1	7.21	28.7	−81.0	2875.9	4476	148.4	110.4	661.9	25.9	131.8	1232.0	418.2	0.34	0.92	1607.0	103.5	201.0	236.8	30.0	15.0	0.0	0.1
Q3	7.41	31.2	4.5	5432.5	8914	262.0	204.0	1380.5	40.4	153.7	2425.5	972.8	0.59	1.76	3032.8	1609.5	673.0	1324.3	66.8	30.6	0.2	0.6
**WW**	**7.4**	**-**	**-**	**350**	**-**	**50**	**7**	**30**	**3**	**200**	**20**	**30**	**-**	**-**	**100**	**15**	**3**	**3**	**0.03**			
**MAC**	**6.5–8.5**	**-**	**-**		**2250**	**-**	**-**	**-**	**-**	**-**	**-**	**-**	**-**	**-**	**20,000**	**10,000**	**5000**	**10,000**	**50**	**-**	**-**	**-**

*WW* worldwide water content of ions (after Langmuir (1997))

*MAC* irrigation water maximum allowable concentration according to USDA (2011)

The measured EC (2198–14,561 µS/cm) indicated that 96% of the studied water showed conductivity values above the permitted limit (2250 µS/cm) for irrigation water. The high EC level resulted from the high concentration of TDS and mineralization of organic materials (Abida & Harikrishna, 2008). The TDS values ranged from 1367 to 8645 mg/l. The high values of salinity can be attributed to the dissolution of limestone, dolomite, and evaporite deposits in the study area as well as the mixing with the trapped ancient seawater (Hassan & Ismail, 2018).

High saline irrigation water can adversely affect soil structure and crop yield. The aridity increases the vulnerability of groundwater and soil to salinity and sodicity hazards, owing to the scarce precipitation, high daily temperatures, and high evaporation rate (Adhanom, 2019). The Eh values ranged from − 213 to + 204 mV with most values in the negative range, indicating anaerobic and reducing conditions within the aquifer. Very high TDS and EC levels are distributed in the central area of the study due to high agriculture and salt lakes activities (Fig. 3b, c). This may be attributed to the change in the lithofacies type, where the marine and evaporite deposits extensively present in the central part of the oasis (Abdel-Mogheeth, 1996; El-Sayed et al., 2017). Besides, the aquifer is subjected to salinization process as indicated by Revelle index, which ranged from 5.1 to 86 (Table 1) by the mixing with the old trapped seawater. This result is in agreement with El-Sayed et al. (2017), who pointed to the mixing of groundwater with the old trapped saltwater.

The relative abundance of the measured ions was in decreasing order: Cl (1917.5 mg/l) > Na (1141.8 mg/l) > SO_4_ (761.1 mg/l) > Ca (201.2 mg/l) > Mg (148.6 mg/l) > HCO_3_ (141.5 mg/l) > K (34 mg/l) > NO_3_ (1.38 mg/l) > NH_4_ (0.46 mg/l). The water type of TCCA is Na-Cl. The prevailing anion in the analyzed groundwater samples is Cl, while the prevailing cation is Na and can be attributed to the mixing with the ancient seawater, dissolution of halite deposits dispersed in the aquifer rocks. The role of the cation exchange process is low and variable through the study area, with most samples showing reverse ion exchange (positive values of CAI-1 and CAI-2) (Table 1).

The sampled groundwater NO_3_ concentrations varied from 0.42 to 3.36 mg/l, with an average value of 1.38 mg/l. The concentration of NH_4_ was around 0.46 mg/l. The concentration of nitrogen ions is low as a result of the confining condition of the aquifer. Generally, NO_3_ in natural groundwater may be up to 10 mg/L, whereas anthropogenic activities like agriculture, septic systems, and animal manure can increase its concentration (USEPA, 2012; Widory et al., 2004). Soil biochemical processes also affect the availability of NO_3_ in groundwater (Stigter et al., 2006).

The mean concentrations of Fe, Mn, Cu, Pb, and Cd were 2359.4, 898.8, 448.9, 865.8, and 52.9 µg/l in the studied groundwater samples were higher than values of the world water (23 times for Fe and 1760 times for Cd). The violation of water quality guidelines is mostly related to human activities (industrial and agricultural) and can cause many health problems (Shah et al., 2021). The presence of high concentrations of Fe and in the carbonate aquifer may be attributed to the water–rock interaction and hydraulic connection with the underline Fe-rich Nubian sandstone aquifer (Abdel-Gawad et al., 2020). Yousif et al. (2016) recorded glauconite and Fe oxides detrital grains within the Miocene carbonate rocks at the northern part of the Western Desert. This is confirmed by the positive correlation (*r* = 0.40) between Fe and Mn (Table 2). Moghra Formation contains about 1.6–36.1%, 0–0.6%, and 5–50 mg/l of Fe_2_O_3_, MnO, and Cu, respectively (Tawfik et al., 2018). This indicates the geogenic source of these elements in the studied water, while Cd and Pb were not recorded in the rocks indicating their anthropogenic source.

**Table 2:**
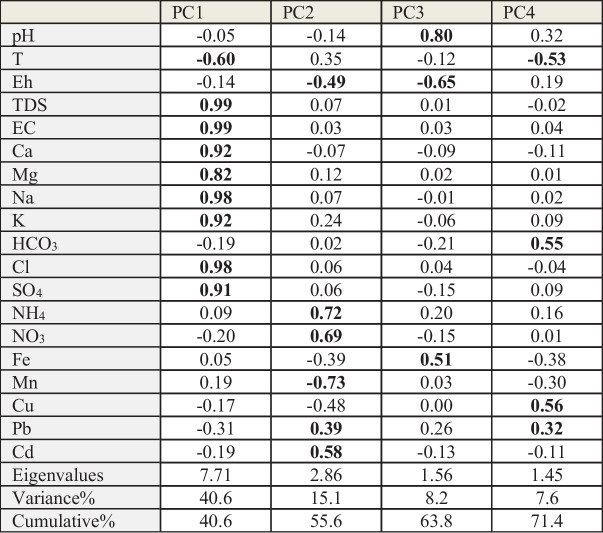
Component matrix of groundwater data

### Temporal variation in salinity

**Fig. 4:**
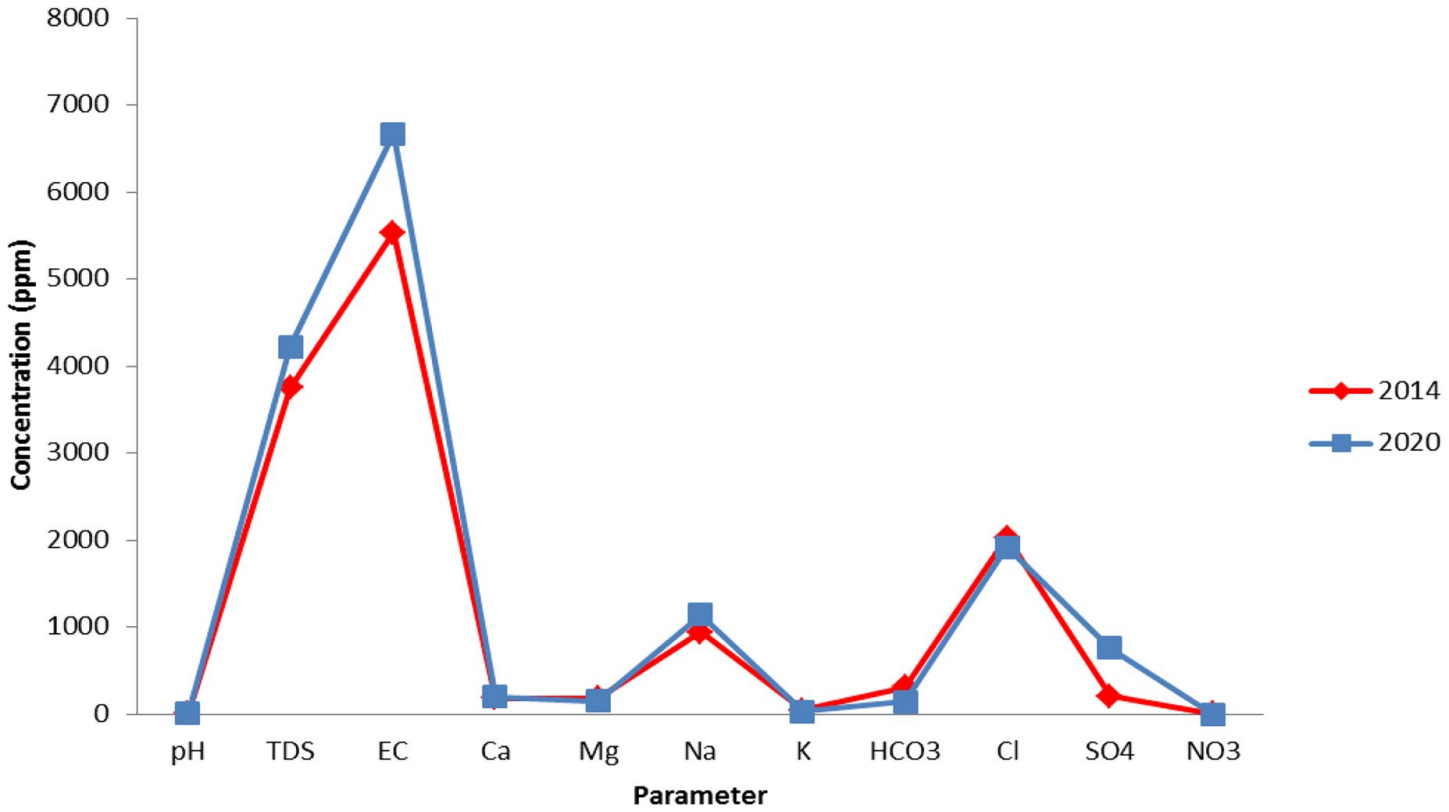
Comparison between Siwa groundwater chemistry in 2014 and 2020

**Fig. 5:**
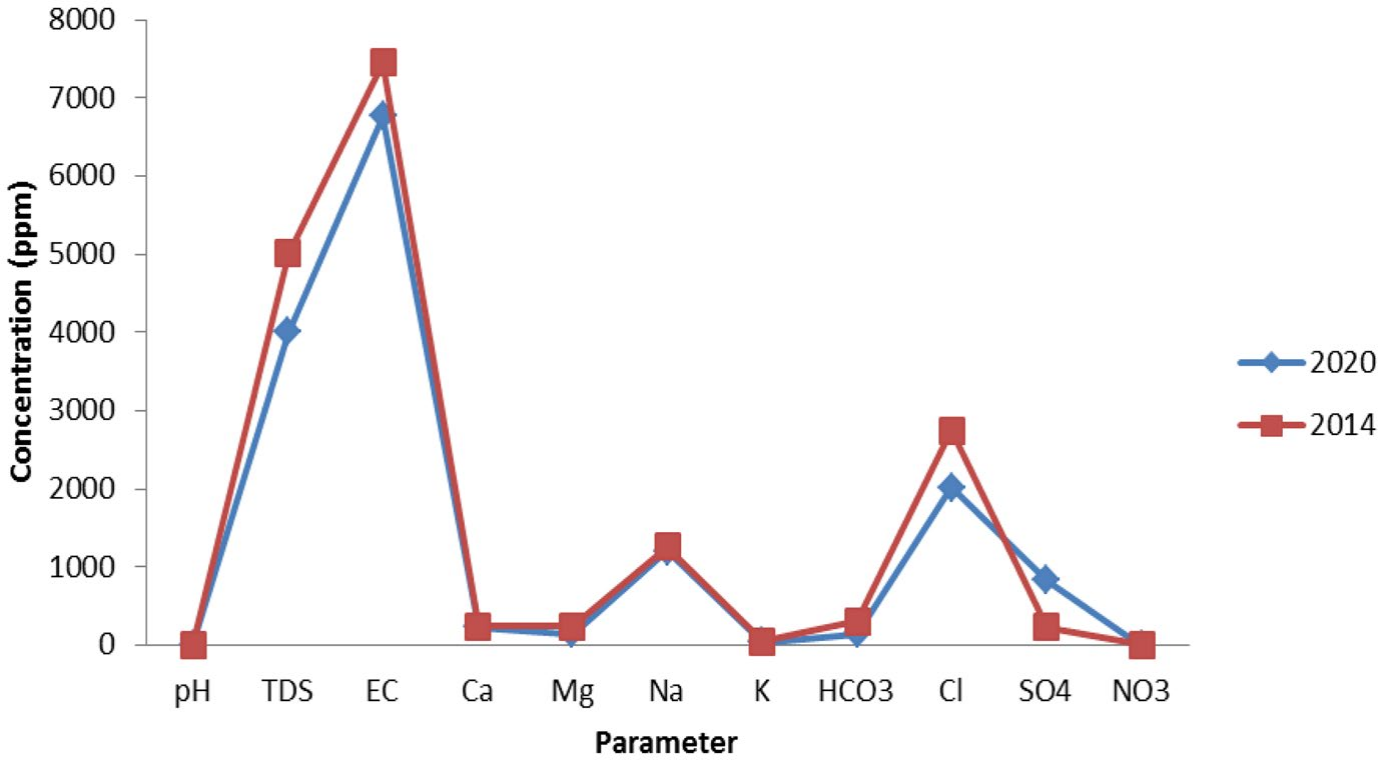
Comparison between Siwa followed up 10 wells chemistry in 2014 and 2020

The comparison of the current hydrochemical results (2020) with the results of 2014 (Salman et al., 2018) indicated a slight variation in TDS and the measured ions (Fig. 4). The TDS has been increased from 3754.3 to 4222.4 mg/l, and the only increased ions are Ca and Na. The collected field data and observation indicated (a) the cession of many illegal wells, (b) control the abstraction of groundwater, and (c) excavation of halite deposits for economic application (construction salt extraction Co.). This has helped in the slight enhancement of groundwater quality and preservation from degradation. Figure 5 illustrated the decrease in TDS and other ions in some wells sampled in 2014 and re-sampled again in 2020. The loss of water from illegal wells that were pumping over the day without any control was about 132,000 m^3^/day. This water was transferred to the lakes, leading to the increase of lakes surface area, degradation of agricultural soil, and formation of salt deposits (Salman et al., 2018). The dramatic increase in salinity from 1996 (4500 µS/cm) to 2013 (10,500 µS/cm) led to an acute decrease in date palm and olive yield (Moghazy & Kaluarachchi, 2020). Therefore, it is important to continue in the environmental preservation rules taken in Siwa for the prevention of this oasis’s unique ecology from degradation.

### Statistical analysis

**Fig. 6:**
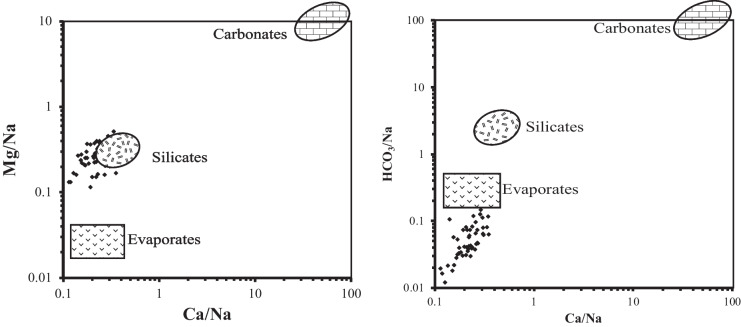
End-member plot for the studied groundwater samples

**Fig. 7:**
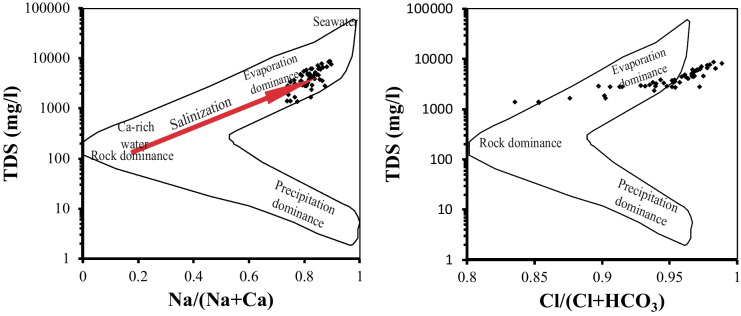
Siwa groundwater plot on Gibbs diagram

Principal component analysis was applied to identify the prevailing hydrochemical processes (Table 2) in the study. The results pointed out the presence of four main principal components (PCs) explain about 71.4% of the total variance of the data set. In the first factor (PC1), which has about 40.6% of the total variance, high positive loadings for salts and major ions, TDS, EC, Ca, Mg, Na, K, Cl, and SO_4_, have been noticed. The loading of these ions in this factor as well as TDS and EC points out the role of salt-bearing sediment (carbonates and evaporates) dissolution in the hydrochemical characteristic of the groundwater. This factor is supported by the end-member (Fig. 6) of Gaillardet et al. (1999) plot, which indicated rock weathering (silicates and evaporates). In addition, the Gibbs (1970) diagram (Fig. 7) illustrated the role of the evaporation process on the water salinity increase under the arid conditions of the study area through a loss of water and concentration of ions formed by water–rock interaction.

The second factor (PC2) represents about 15.1% of the total variance, showing negative loading of Eh and Mn, as well as positive loading of NO_3_, NH_4_, Pb, and Cd suggesting anthropogenic activity as the source of N, Pb, and Cd. Agricultural activities can contribute to NO_3_ and NH_4_ ion concentrations in the groundwater. The drainage water at Siwa contains about 66.2 mg/l NO_3_ (Hedia, 2015). This also indicates the role of the reducing environment on the Mn levels. The third factor (PC3), which has about 8.2% of the total variance, is showing negative loading of Eh against pH and Fe suggesting that the redox process in the study area is the main controller of Fe geochemistry. The fourth (PC4) has about 7.6% of the variances and is mainly participated by HCO_3_, Cu, and Pb in negative correlation with T and is possibly related to infiltration of these ions from the surface through the structure lineaments. Abdel-Gawad et al. (2020) pointed out the structural lineaments (faults and/or joints) in the transfer of polluted water from the surface drain into the carbonate aquifer.

### Hydrochemical facies

**Fig. 8:**
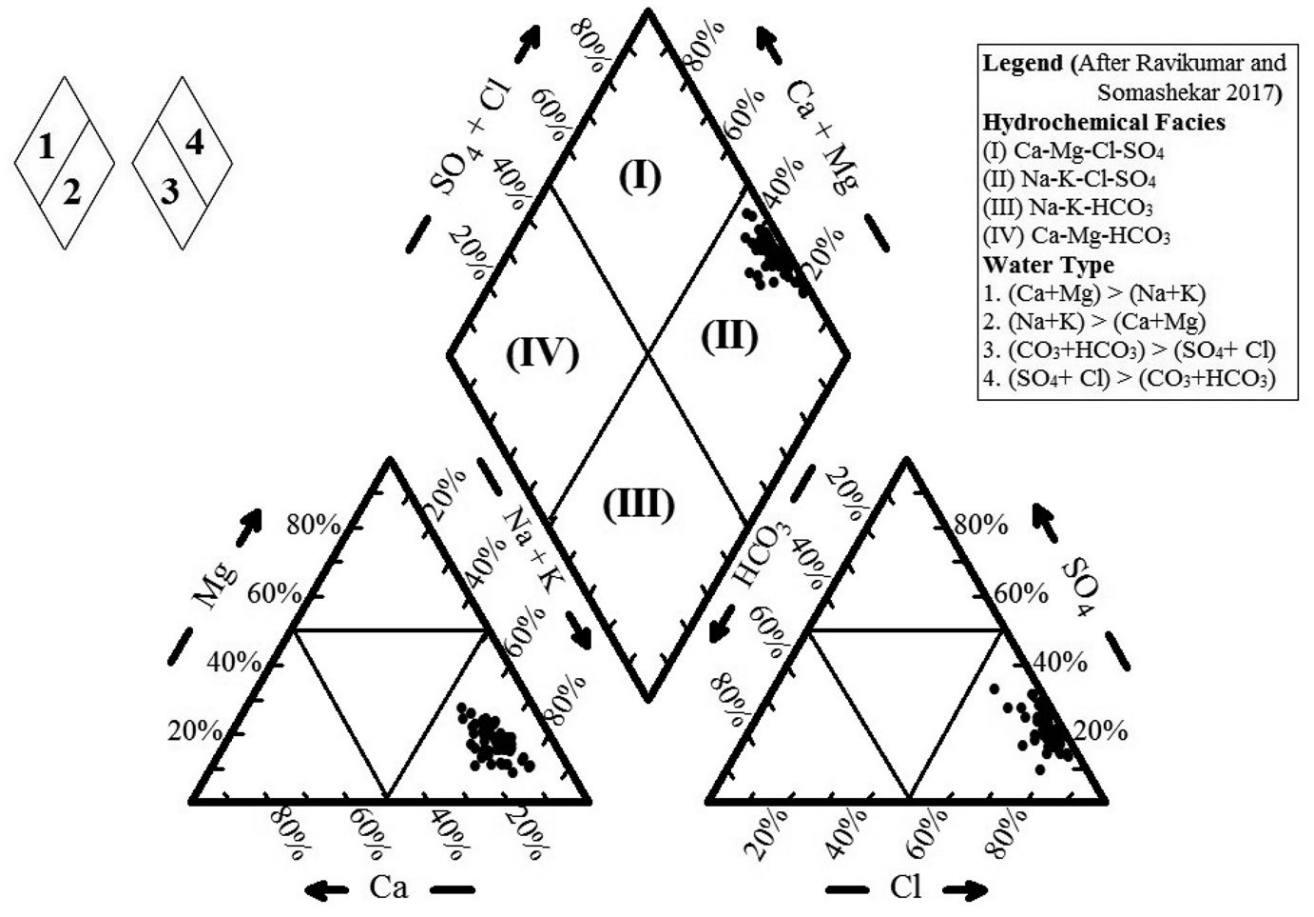
Piper diagram for classification of groundwater of Siwa area

To understand the hydrogeochemical dominance in the studied groundwater, the trilinear diagram of Piper (1944) was used (Fig. 8). In the left triangle (the cation plot field), the entire groundwater samples were plotted in the alkali (Na + K) sector indicated by the dominant alkali cations. On the other hand, in the right triangle (the anion plot field), all the samples plotted fall in the Cl sector. The diamond shape plot shows that the samples are in field II (Na–K–Cl–SO_4_ type) indicating the prevailing of alkalis (Na + K) and stronger acidic anions (Cl + SO_4_) over the alkaline earths (Ca + Mg) and weaker acidic anions (CO_3_ + HCO_3_). The samples are clustered at seawater facies, indicating their high salinity as well as halite weathering (Kaur et al., 2019). The predominance of Na in the groundwater samples may be attributed to silicate mineral weathering and ion exchange processes on clay particles (Herojeet et al., 2017).

#### Evaluation of water quality for irrigation

**Fig. 9:**
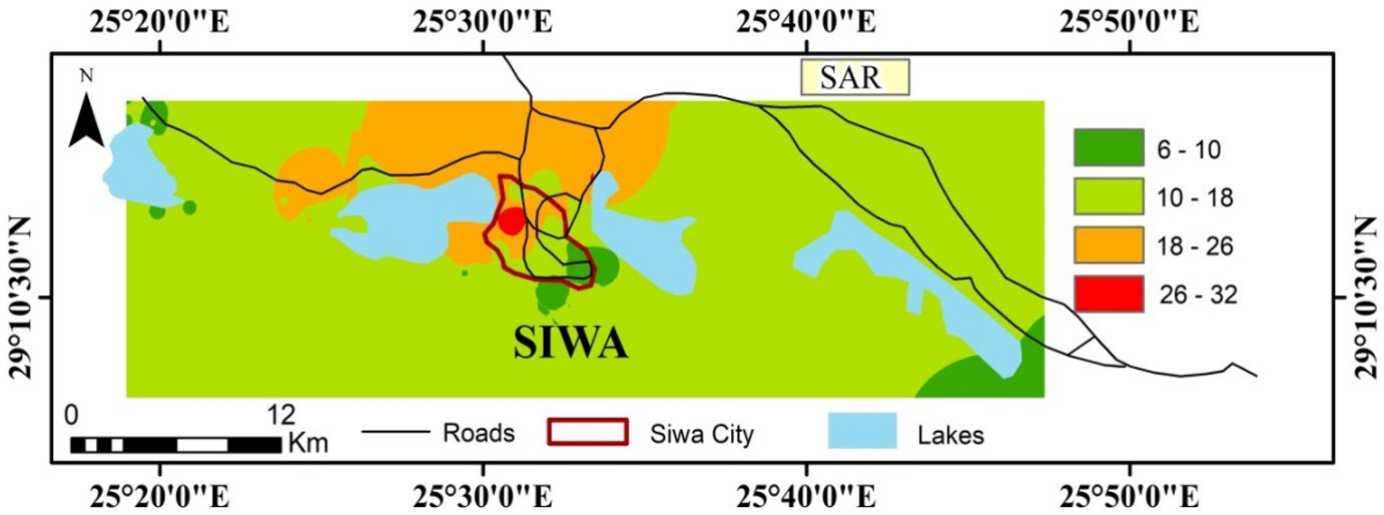
Distribution of sodium absorption ratio (SAR) for the study area

Among the most important factors that affect the suitability of water for irrigation are salinity, sodium hazard, and toxic elements (Elnazer & Salman, 2021; Elnazer et al., 2021; Loh et al., 2020). An excess of salt leads to a difference in osmotic pressure around the roots of the plants, which leads to the plant’s inability to absorb water and the nutrients it carries, causing the plants to wilt and dry up. The salinity can be expressed using electrical conductivity. Water with electrical conductivity of less than 2250 µS/cm is considered acceptable for irrigation (Richards, 1954). The presence of high Na concentration in irrigation water is linked with soil property deterioration and water infiltration reduction as a result of soil coagulation (Gupta, 2012; Todd & Mays, 2005). Sodium hazard can be assessed by the calculation of the sodium adsorption ratio (SAR). In geochemical regard, the excess of Na in irrigation water over Ca and Mg, the cation exchangers, will lead to the saturation with Na and hence the dispersion of the clay particles and consequentially destruction of soil structure (Venkateswaran & Vediappan, 2013; Zaman et al., 2018). Based on SAR classification, 18 (33%) groundwater samples are categorized as excellent water for irrigation purpose, 25 (46%) samples are categorized as good water for irrigation purpose, 8 (15%) samples are categorized as doubtful water for irrigation purpose, and only 3 (6%) samples are unsuitable for irrigation (Table 3). The wells producing water contains high Na concentration can cause harmful levels of exchangeable Na in most soil types and requires special soil treatment as the construction of good drainage system. The distribution map of SAR shows that the higher values are located in the center of the study area near Siwa city and near the agriculture activity (Fig. 9).

**Table 3:** Classification of irrigation water based on SAR values

SAR value	Class	Studied samples	No. of samples	Percentage of samples
≤ 10	Low sodium hazard (S1)	7, 11–14, 20, 21, 23, 24, 29, 30, 36–38, 51–54	18	33
10–18	Medium sodium hazard (S2)	1, 4, 8–10, 15, 18, 19, 22, 25–28, 31–35, 39, 42, 44–46, 49, 50	25	46
18–26	High sodium hazard (S3)	5, 6, 16, 17, 40, 41, 43, 48	8	15
> 26	Very high sodium hazard (S4)	2, 3, 47	3	6

**Fig. 10:**
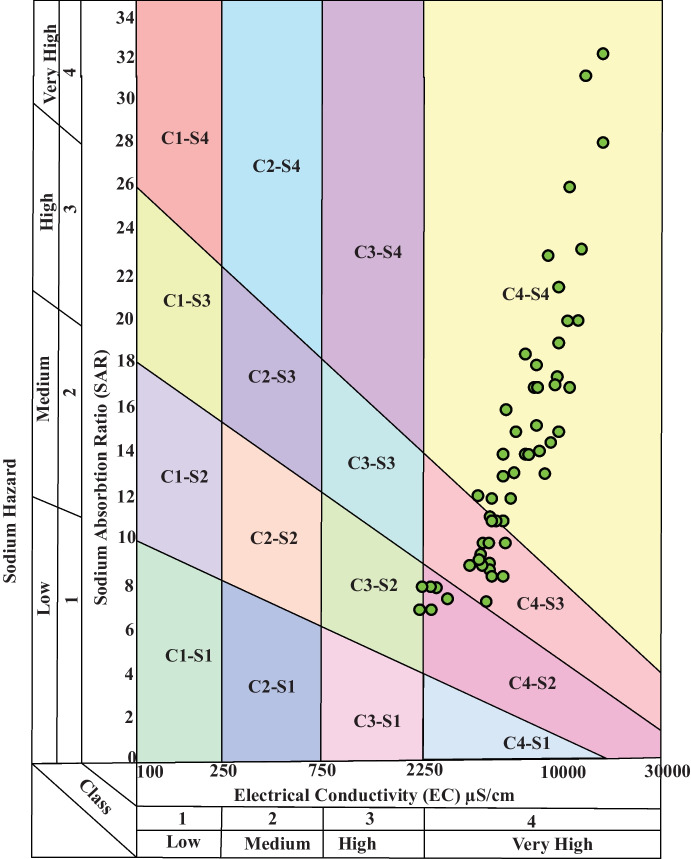
USSL’s diagram for the suitability of water for irrigation

The USSL (Richards, 1954) has merged plotted EC vs SAR on one diagram, due to their importance to the irrigation process that can divide irrigation water into 16 classes. The USSL diagram (Fig. 10) locates Siwa water in the C4-S4, C4-S3, C4-S2, and C3-S2 classes with 61, 28, 7, and 4% of the wells, respectively. It appears that most of the studied wells 89% are located in the very high saline water with EC > 2250 µS/cm, which can be harmful to most crops except salt-tolerant ones. Special procedures are needed to control the salinity hazard including modern irrigation techniques, planting suitable crops, and leaching and adequate drainage system.

Despite the recorded high concentrations of Fe and Mn in the studied water, they are still within the permissible limits for irrigation. The danger in these waters comes from the presence of high concentrations of cadmium in about 50% of the studied plants above the permissible limits for irrigation. The presence of cadmium is very dangerous to plants, as it causes plant metabolism disruption, seed germination, shoot, and root reduction reduces nutrients translocation and uptake, and inhibits plant morphology and physiology (Haider et al., 2021). The bioaccumulation of cadmium in plants may reach the higher food chain and then humans, causing health problems (Elnazer & Salman, 2021; Salman et al., 2019; Seleem et al., 2021).

## Conclusion

Groundwater is extracted from the shallow limestone aquifer in Siwa Oasis for use in irrigation. The prevailing ions in this groundwater are Na and Cl. The change in lithofacies of rock-bearing water controls the chemistry of water. The central part of the oasis has the highest TDS value inside with Ca, Mg, Na, K, SO_4_, and Cl. There are four factors controlling water chemistry: water–rock interaction, agricultural activities, the Redox process, and infiltration of surface water. The hydrologic and environmental actions in the last years have had a noticeable impact on groundwater quality. The salinity and presence of Cd in some samples make this water hazardous for irrigation. The construction of desalination stations is necessary for protecting the oasis from the hazard of soil degradation and plant yield decrease.

## Data Availability

All data generated or analyzed during this study are included in this published article (and its supplementary information files).
